# Potential Molecular Interactions and In Vitro Hyperthermia, Thermal, and Magnetic Studies of Bioactive Nickel-Doped Hydroxyapatite Thin Films

**DOI:** 10.3390/ijms26031095

**Published:** 2025-01-27

**Authors:** Muhammad Sohail Asghar, Uzma Ghazanfar, Muhammad Rizwan, Muhammad Qasim Manan, Athar Baig, Muhammad Adnan Qaiser, Zeenat Haq, Lei Wang, Liviu Duta

**Affiliations:** 1 Ministry of Education Key Laboratory of Green Preparation and Application for Functional Materials, School of Materials Science and Engineering, Hubei University, Wuhan 430062, China; sohail.asghar673@gmail.com (M.S.A.); wanglei1430635091@163.com (L.W.); 2 Department of Physics, University of Wah, Wah Cantt 47040, Pakistan; uzma.ghazanfar@uow.edu.pk (U.G.); m.rizwan.phy@gmail.com (M.R.); 3 Department of Mechatronics Engineering, University of Wah, Wah Cantt 47040, Pakistan; q.manan796@gmail.com; 4 Department of Electrical Engineering, University of Engineering and Technology, Lahore 54890, Pakistan; atharbaiguet@gmail.com; 5 College of Physics and Optoelectronic Engineering, Shenzhen University, Shenzhen 518060, China; m.adnan.qaiser@gmail.com; 6 Department of Biosciences, University of Wah, Wah Cantt 47040, Pakistan; zeenat.haq@uow.edu.pk; 7 Lasers Department, National Institute for Laser, Plasma and Radiation Physics, 409 Atomistilor Str., 077125 Magurele, Romania

**Keywords:** nickel-doped hydroxyapatite (Ni:HAp) thin films, HDOCK simulation, in vitro testing, magnetic hyperthermia, varying number of laser pulses, pulsed laser deposition technique

## Abstract

The treatment of bone cancer often necessitates the surgical removal of affected tissues, with artificial implants playing a critical role in replacing lost bone structure. Functionalized implants represent an innovative approach to improve bio-integration and the long-term effectiveness of surgery in treating cancer-damaged bones. In this study, nickel-substituted hydroxyapatite (Ni:HAp) nanoparticles were deposited as thin films using laser pulses in the range of 30,000–60,000. Comprehensive structural, infrared, optical, morphological, surface, and magnetic evaluations were conducted on the synthesized Ni:HAp thin films. The magnetic hysteresis (M-H) loop demonstrated an increase in the saturation magnetization of the films with a higher number of laser pulses. A minimum squareness ratio of 0.7 was observed at 45,000 laser pulses, and the M-H characteristics indicated a shift toward ferromagnetic behavior, achieving the desired thermal response through an alternating magnetic field application within 80 s. Thermogravimetric analysis revealed distinct thermal stability, with the material structure exhibiting 46% degradation at 800 °C. The incorporation of bioactive magnetic nanoparticles in the thin film holds significant promise for magnetic hyperthermia treatment. Using HDOCK simulations, the interactions between ligand molecules and proteins were also explored. Strong binding affinities with a docking score of −67.73 were thus observed. The presence of Ca^2+^ ions enhances electrostatic interactions, providing valuable insights into the biochemical roles of the ligand in therapeutic applications. Intravenous administration of magnetic nanoparticles, which subsequently aggregate within the tumor tissue, combined with an applied alternating magnetic field, enable targeted heating of the tumor to 45 °C. This focused heating approach selectively targets cancer cells while preserving the surrounding healthy tissue, thereby potentially enhancing the effectiveness of hyperthermia therapy in cancer treatment.

## 1. Introduction

Nanomaterials have garnered significant attention for their diverse characteristics, establishing them as a pivotal class of materials in developing innovative devices. Such devices find applications across diverse fields, including physics, biomedicine, biology, and pharmaceuticals [1,2]. Nano-crystallites, which are aggregates of nanoparticles with a highly ordered atomic arrangement, exhibit a unique discontinuity in electronic energy levels. These structures, commonly known as “quantum dots” or “artificial atoms”, are typically composed of specific semiconductor materials [3,4] and offer exceptional potential for advanced technological applications. Due to their simultaneous magnetic and electrical behavior, which can be controlled through particle size and density modification, the synthesis and characterization of magnetic nano-aggregates have raised a great deal of attention in the present era of technology [5,6].

Hydroxyapatite (HAp) is renowned for its structural resemblance to the mineral phase of bone and teeth, which grants it exceptional biocompatibility and osteoconductivity [7]. These inherent properties have established HAp as a cornerstone material in applications such as bone tissue engineering, dental implants, and drug delivery systems [8]. To meet the rising demand for multifunctional materials, research has increasingly focused on modifying HAp to enhance its magnetic, thermal, and catalytic properties. A prominent approach in this domain is the doping of HAp with transition metals, such as nickel (Ni) [9]. Nickel’s ferromagnetic properties significantly enhance the functionality of HAp, enabling its use in advanced biomedical and technological fields. For instance, Ni-doped HAp (Ni:HAp) holds potential for magnetic hyperthermia in cancer treatment, where precise heat generation is crucial for therapeutic efficacy. Additionally, its enhanced magnetic properties make it suitable for magnetic resonance imaging (MRI), providing contrast and aiding in diagnostics, as well as advancing tissue engineering applications [10,11].

Inflammation frequently occurs as a result of uncontrolled cellular proliferation, which can lead to tumor formation when a single cancerous cell undergoes unregulated and accelerated division. Tumors compete with healthy tissues for essential nutrients derived from the bloodstream, thereby disrupting normal organ function [12]. Processes such as angiogenesis and metastasis not only promote the rapid growth and spread of tumors but also pose significant challenges to treatment, complicating therapeutic strategies and leading to poorer patient outcomes. Effectively targeting these mechanisms is crucial for advancing cancer therapies, improving treatment efficacy, and enhancing patient survival rates [13]. The projected global burden of cancer, with over 20 million new cases and 13.2 million cancer-related deaths expected by 2030 [14], has driven extensive research to combat this deadly disease [15,16,17,18]. Chemotherapy and radiation therapy currently serve as the gold standards for treatment. Additionally, localized temperature elevation techniques, such as ultrasound, radio waves, nuclear radiation, microwaves, and hot water, have been employed to enhance therapeutic outcomes [19]. However, increasing attention is being directed toward novel complementary treatments, including hyperthermia [20,21]. Thus, HAp-coated iron oxide nanoparticles (Fe_2_O_3_ NPs), when combined with magnetic hyperthermia, were proposed as a safe and effective therapeutic approach for various types of cancer treatment [22]. In a complementary study [23], HAp-functionalized γ-Fe_2_O_3_ NPs demonstrated excellent biocompatibility with no detectable toxicity, highlighting their potential application in magnetic hyperthermia therapy for cancer and bone regeneration.

Hyperthermia involves elevating the temperature of the tumor microenvironment to a range of 43 °C to 45 °C, a level at which cancerous cells are effectively destroyed while normal healthy cells remain largely unaffected. This selective efficacy stems from the inherent differences between cancerous and healthy tissues. Tumor cells exhibit a compromised vascular network with fewer blood vessels compared to normal cells, resulting in stasis, hemorrhages, and vascular occlusion when subjected to thermal stress at approximately 43 °C. However, temperatures exceeding 45 °C can cause damage to healthy cells, underscoring the need for precise temperature control during hyperthermia therapy [24].

**Figure 1:**
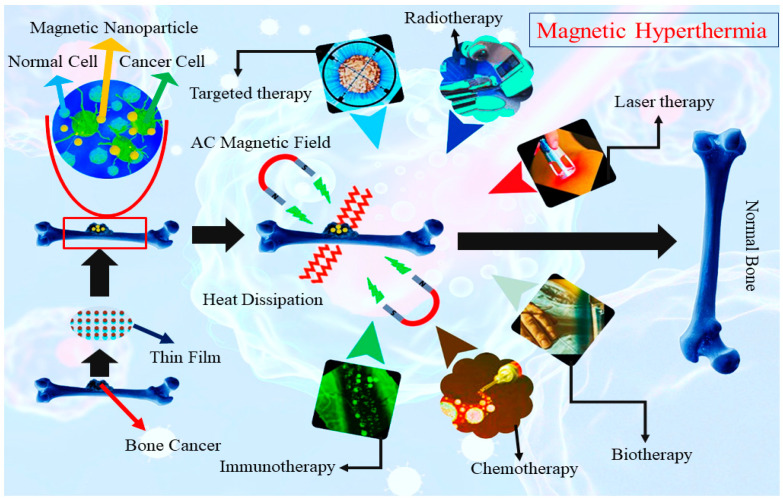
Proposed mechanism of magnetic hyperthermia for bone cancer treatment.

With the rapid development of nanotechnology, magnetic materials have emerged as a promising strategy for delivering localized hyperthermia [25]. Magnetic biomaterials, such as silicon thin films integrated into affected areas, can effectively treat conditions like bone cancer. When exposed to an external magnetic field, these thin magnetic films generate heat due to magnetic hysteresis loss, as illustrated in Figure 1. The fabrication of such ultra-thin films poses challenges, particularly in achieving the desired microstructure, composition, and electrical and magnetic properties. Additionally, most reports highlight the absence of robust and reproducible synthetic strategies [22]. While conventional deposition techniques, such as sol–gel and spray pyrolysis, are valuable for certain applications, they often face challenges in achieving homogeneous films with precise thickness control. These drawbacks can significantly compromise the films’ functional properties, particularly in biomedical applications where consistent bioactivity and mechanical stability are essential. However, advancements in laser surface modification techniques, including radio frequency magnetron sputtering [26] and pulsed laser deposition (PLD), have demonstrated effective incorporation of dopants into the HAp matrix and a significant improvement of interfacial adhesion in metal–ceramic systems such as HAp on titanium [27,28,29,30].

Currently, molecular docking has become a highly effective and cost-efficient method for the design and evaluation of drugs and biomaterials intended for cancer or tumor treatment [31]. This approach provides detailed insights into the interactions between drug candidates and their target receptors, facilitating the prediction of binding affinities and mechanisms of action [32]. Moreover, by non-covalently positioning a molecule within the binding site of a macromolecular target, molecular docking enables a detailed examination of binding interactions, providing accurate information about the specific active sites involved in ligand–target interactions [33]. This precision makes it an indispensable tool in the rational design of therapeutic agents.

The limited exploration of in vitro hyperthermia mechanisms in doped HAp materials presents significant challenges for their effective application in biomedical therapies, notably cancer treatment. Many studies have predominantly focused on the synthesis and characterization of these materials, emphasizing a single property (e.g., magnetic, or thermal). Integrating comprehensive investigations of molecular interactions, thermal/magnetic behaviors, and in vitro hyperthermic effects, which are critical for the development of doped-HAp-based materials for biomedical applications like cancer therapy, is often overlooked [34]. This gap in research (between material design and biomedical application) hinders the optimization of parameters crucial for inducing controlled hyperthermia in target tissues. For instance, while Kurinjinathan et al. [35] synthesized Ni:HAp nanoparticles and analyzed their structural and morphological properties, their study did not extend to evaluating their performance in hyperthermia applications, leaving a critical aspect of their biomedical potential unexplored. Additionally, while the magnetic properties of iron-doped HAp have been explored for hyperthermia applications, insufficient attention to the material’s biocompatibility and interaction with biological systems can lead to suboptimal therapeutic outcomes [10]. In contrast, recent research has begun to address these shortcomings by integrating in vitro hyperthermia assessments with biological studies. Tithito et al. [36] developed a composite material combining trace element co-doped HAp with Mn–Zn ferrite nanoparticles, termed THAiBioMags. Their comprehensive evaluation encompassed magnetic hyperthermia performance, drug delivery capabilities, and biocompatibility, including bone cell adhesion and zebrafish assays. The study demonstrated that THAiBioMags exhibited a specific absorption rate of 9.44 W g^−1^ under an alternating magnetic field, indicating effective heat generation suitable for hyperthermia applications. Moreover, the material showed sustained drug release and excellent biocompatibility, underscoring the importance of multifaceted analyses in developing functional biomaterials for cancer therapy.

In this work, nanoparticles were synthesized and subsequently deposited as thin films (further noted as Ni:HAp thin films) on silicon (Si) substrates using the PLD technique. The depositions were carried out at a substrate temperature of 500 °C in a vacuum environment, with the number of laser pulses varied between 30,000 and 60,000. The deposition of Ni:HAp thin films within this specific range represents a novel approach designed to investigate the influence of the number of laser pulses on the properties of the fabricated films, with a particular focus on variations in thickness and surface quality. The magnetic properties of the deposited films, i.e., coercive field, saturation magnetization, and remanence ratio, were characterized. In this study, we also examine the interactions between ligand molecules and proteins, with a focus on their binding affinities and electrostatic properties. Through the application of docking analyses and electrostatic mapping, we identify critical factors that influence ligand–protein interactions. Our findings emphasize the pivotal role of charge distributions and the potential involvement of ion interactions, such as those mediated by Ca^2+^ ions, in enhancing the stability and affinity of binding interactions. These insights contribute to a deeper understanding of the molecular mechanisms underlying ligand–protein binding.

## 2. Results

### 2.1. Heat Generation and Thermogravimetric Analysis

#### 2.1.1. Heat Generation Assessment

**Figure 2:**
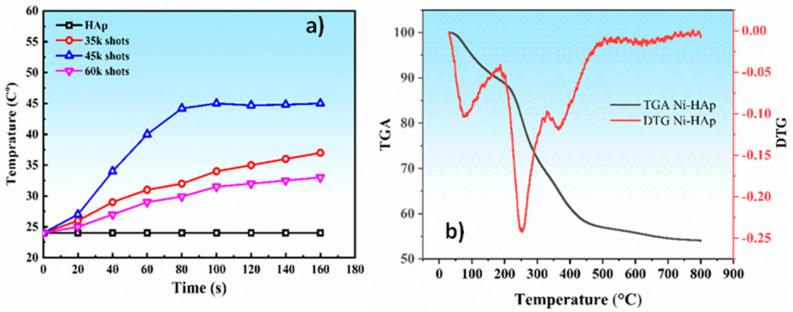
Thermal behavior of Ni:HAp materials: (**a**) heat generated by Ni:HAp thin films deposited on Si substrates, under different numbers of laser pulses, measured in agar solutions subjected to an alternating magnetic field; (**b**) thermogravimetric curve of Ni:HAp nanopowders, showing weight changes as the material is heated from 30 °C to 800 °C.

The heat variations over time for agar gels containing Ni:HAp thin films in an alternating magnetic field of 300 Oe at 100 kHz are illustrated in Figure 2a. The temperature of the agar gels increased progressively over time, demonstrating consistent heat generation by the magnetic Ni:HAp thin films. Among the investigated samples, the Ni:HAp thin films prepared with 45,000 laser pulses, which exhibited the lowest squareness ratio, showed superior performance. The temperature of these samples rose above 45 °C within just 80 s, which is a known critical threshold for effective hyperthermia applications [37].

#### 2.1.2. Thermogravimetric Analysis

Figure 2b presents the thermogravimetric analysis (TGA) curve of Ni:HAp nanopowder, heated from 30 °C to 800 °C. Between 40 °C and 220 °C, the nanopowder experienced a 12% weight loss, which likely corresponds to the removal of adsorbed water and surface-bound hydroxyl groups. Beyond 220 °C, a gradual weight decrease was observed up to 450 °C, where an inflection point marked a more significant decomposition process. Over the 220 °C to 450 °C range, the nanopowder exhibited an additional 30% weight loss, indicating the degradation of organic residues or structural rearrangements. As the temperature increased further to 800 °C, the weight of Ni:HAp decreased slightly, with only a 4% weight loss observed before reaching thermal stability.

The thermal events observed in the differential thermal analysis (DTA) curve indicate the removal of water molecules and other thermal transitions occurring within the material’s structure. Three primary thermal events were identified:

(i) Surface water evaporation: The first thermal transition, occurring around 77 °C, corresponds to the evaporation of water molecules adsorbed onto the material’s surface. This process involves the removal of loosely bound water.
(ii) Loss of structural water: The second transition, observed at approximately 252 °C, corresponds to the loss of structural water that is chemically bound within the nanopowder. This represents a deeper alteration of the material compared to surface water removal.
(iii) Chemical reaction and formation of calcium pyrophosphate: The third event, occurring at 365 °C, involves a chemical reaction between two molecules of calcium monohydrogen phosphate, resulting in the formation of calcium pyrophosphate and water. This process reflects a structural transformation within the material.

At 800 °C, the TGA curve reveals a significant mass loss of ~46%. This significant mass loss is likely due to the thermal degradation of the material, involving the release of volatile components and structural decomposition. These findings offer valuable insights into the thermal stability and decomposition behavior of the material, which are critical for evaluating its potential applications under high-temperature conditions.

### 2.2. X-Ray Diffraction (XRD) Measurements

**Figure 3:**
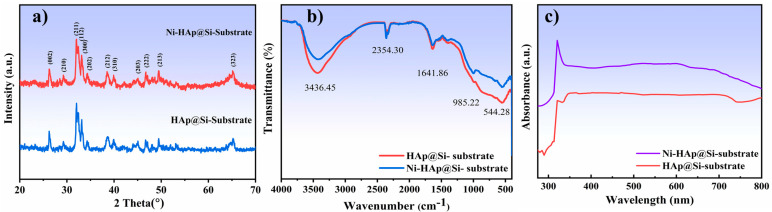
Comparative analysis of HAp and Ni:HAp thin films deposited on silicon substrates: (**a**) XRD patterns, (**b**) FTIR spectra, and (**c**) UV-Vis diffuse reflectance spectra (DRS).

The XRD spectra of the HAp and Ni:HAp thin films deposited on Si substrates (further denoted as HAp@Si-Substrate and Ni:HAp@Si-Substrate) are shown in Figure 3a. Both thin films exhibit a hexagonal crystal structure, with prominent diffraction peaks observed at 2θ = 26.28°, 29.25°, 32.06°, 32.40°, 33.17°, 34.28°, 38.54°, 40°, 45.10°, 46.80°, 49.52°, and 65.20°. These peaks correspond to the (002), (210), (211), (112), (300), (202), (212), (310), (203) (222), (213), and (323) crystallographic planes, indicating a well-defined orientation within the material’s lattice structure.

The XRD patterns of the samples indicate that the observed diffraction peaks are consistent with the standard data indexed in Joint Committee on Powder Diffraction Standards (JCPDS) Card No. 09-0432, corroborating findings from previous studies. The absence of any additional peaks confirms the lack of contaminant phases such as silicon oxide or other calcium phosphate (CaP) species [38]. At a first-order approximation, the substitution of Ni into HAp does not significantly alter the diffraction pattern, suggesting that the doping likely occurs at the interstitial sites of the HAp crystal lattice on the Si substrate. Using the Scherer formula, the crystallite size of the sample was estimated to be approximately 38 nm, highlighting the material’s nanoscale structural characteristics.

### 2.3. Fourier Transform InfraRed Spectroscopy (FTIR) Analysis

Reports from the literature [39] demonstrated that the incorporation of Si into the Ni:HAp lattice significantly altered the transmittance bands. Thus, the inclusion of Si influenced the HAp structure, particularly affecting the vibrational bands of the P–O groups. This is also the case in the current study, as shown in Figure 3b. The primary structural changes observed with the incorporation of Ni in hydrothermal particles are the marked reductions in the intensities of the hydroxyl band at ~3436 cm^−1^, the band at ~544 cm^−1^, and the phosphate stretching band at ~985 cm^−1^. These structural variations are attributed to the replacement of (PO_4_)^3−^ units with (SiO_4_)^4−^ tetrahedra within the HAp structure. This substitution induces a decrease in the number of hydroxyl groups to balance the additional negative charge introduced by the incorporated silicate groups. Consequently, vacancies are formed in the (OH)^−^ sites, as described by the reaction (PO_4_)^3−^ + (OH)^−^ → (SiO_4_)^4−^ + V(OH), where V(OH) represents hydroxyl vacancies. The observed FTIR activity aligns with the substitution mechanism, corroborating the formation of (SiO_4_)^4−^ tetrahedra and their coherent interaction with (PO_4_)^3−^ groups in HAp [39]. This substitution accounts for the observed alterations in the vibrational modes, confirming the structural incorporation of Ni nanoparticles into the HAp phase.

### 2.4. UV-Vis Outcomes

UV–Vis spectrophotometric analysis is employed to monitor and confirm the formation of nanoparticles. The initial findings indicate that small, spherical nanoparticles exhibit a surface plasmon resonance (SPR) band extending within the 300–500 nm range, with a peak centered around 320 nm. Figure 3c presents the UV-Vis diffuse reflectance spectra (DRS) of Ni:HAp thin films fabricated by the PLD technique. The absorption spectra of Ni:HAp reveal an absorption band with a maximum between 310 and 330 nm, characteristic of the plasmon resonance associated with metallic nanoparticles [40,41]. This phenomenon is influenced by factors such as particle size, loading, shape, and the surrounding environment.

The increased shift in the absorption band edge confirmed the successful incorporation of Ni^2+^ ions into the Ca^2+^ site within the HAp structure. It is important to mention that the Ni^2+^ ions are responsible for generating the characteristic absorption. This finding underscores the role of Ni in modifying the optical properties of the HAp-based material.

### 2.5. Morphology and Energy-Dispersive X-Ray Spectroscopy Investigations

**Figure 4:**
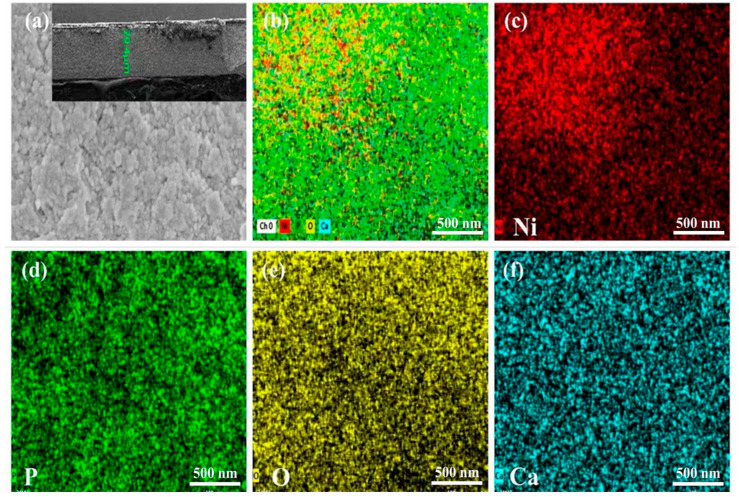
(**a**) Field emission scanning electron microscopy image of Ni:HAp thin films and (**b**–**f**) energy-dispersive spectroscopy elemental mapping of Ni:HAp thin film key elements (i.e., Ca, O, P, and Ni).

Field emission scanning electron microscopy (FE-SEM) provides insights into the morphology of Ni:HAp thin films, while energy-dispersive X-ray spectroscopy (EDS) mapping offers a detailed elemental analysis of their composition. As shown in Figure 4a, the Ni:HAp thin film exhibits a uniform deposition on the surface, with a thickness of ~29.4 μm (as indicated in the inset of Figure 4a).

The EDS mapping (Figure 4b–f) confirms the prominent presence of key elements, including calcium (Ca), oxygen (O), phosphorus (P), and nickel (Ni), uniformly distributed across the thin film’s surface. This elemental distribution aligns with the Ni:HAp crystal lattice, demonstrating the successful incorporation of Ni into the HAp structure.

Figure 4b–f and Table S1 provide detailed individual elemental maps and compositions, indicating the distribution of key components within the HAp structure. Calcium, as the primary cationic component, forms the structural backbone of HAp, while phosphorus contributes to the phosphate groups crucial for bone mineralization. Oxygen, which constitutes a significant proportion of the HAp composition, is distributed throughout the matrix, playing a vital role in stabilizing the crystal lattice. Nickel, introduced as a dopant, is uniformly dispersed within the HAp matrix, as evidenced by its homogeneous distribution across the EDS maps. This even distribution indicates the successful incorporation of Ni into the HAp lattice without phase separation or clustering. The elemental mapping and compositional data emphasize the homogeneity and purity of the Ni:HAp thin film, reinforcing its structural integrity.

The combination of FE-SEM and EDS analyses thus validates the material’s structural and compositional integrity, which is crucial for its intended applications.

### 2.6. Atomic Force Microscopy (AFM) and Magnetic Study of Nickel/Hydroxyapatite Thin Films

#### 2.6.1. AFM Investigations

**Figure 5:**
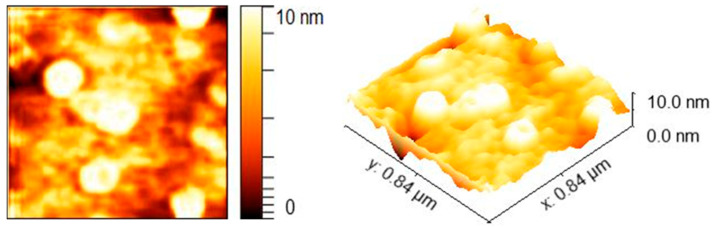
AFM image of the particulates present on the surface of Ni:HAp thin films (**left image**), and 3D image of the particulates present on the surface of Ni:HAp thin films (**right image**).

An atomic force microscope equipped with a sharpened silicon tip with a radius of less than 10 nm was utilized to examine the shape and size of the grains. Surface profile images were acquired by operating the AFM in tapping mode, with a scan size of 0.84 µm and a scan rate of 1 Hz. Notably, the particulates present on the surface of Ni:HAp thin films exhibit an intriguing doughnut-like morphology, as illustrated in Figure 5.

Line analysis of the AFM data reveals that these particulates have an average height of less than 2 nm and an average width of ~20 nm, as depicted in Figure S1.

#### 2.6.2. Magnetic Studies

**Figure 6:**
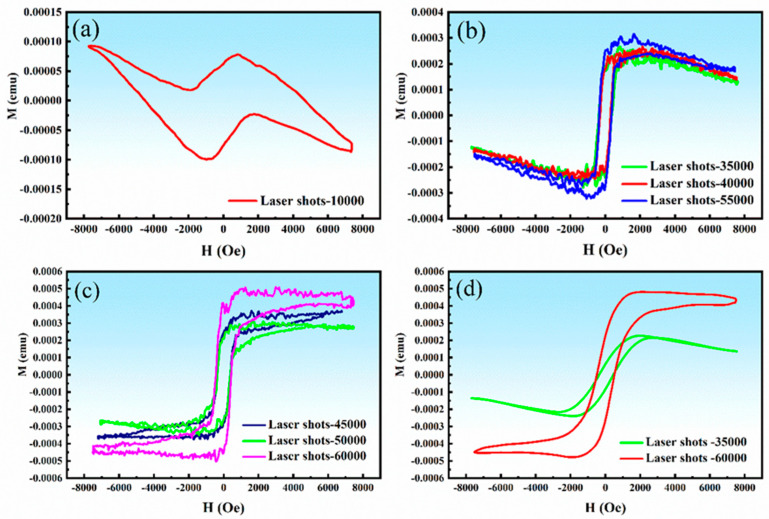
Magnetic hysteresis (M-H) curves recorded after (**a**) initial 10,000; (**b**) 35,000, 40,000, and 55,000; (**c**) and 45,000, 50,000, and 60,000 laser pulses, and (**d**) magnetization shift observed as the number of laser pulses increased from 35,000 to 60,000.

Figure 6 presents the magnetization (M-H) curves of Ni:HAp@Si-Substrates prepared with varying laser pulses (i.e., 35,000–60,000) at RT. The magnetization of all samples increased rapidly with the applied magnetic field and then plateaued, exhibiting characteristics of ferromagnetic behavior.

With an increasing number of applied laser pulses, the magnetization of the specimens increased significantly, reflecting the influence of the dopant. This could be attributed to a few factors, among which one could mention: (i) enhanced dopant incorporation and redistribution and (ii) the formation of magnetic phases or nanostructures. In the first case, laser pulses can generate localized high temperatures, leading to partial melting, diffusion, and recrystallization of the surface layer. This promotes better incorporation and redistribution of dopant ions (e.g., Ni) into the crystal lattice of the material, amplifying the material’s overall magnetic properties. In the second case, the application of a higher number of laser pulses can induce phase transformations or the formation of dopant-rich magnetic clusters or nanoparticles. Rapid cooling after laser irradiation can trap these clusters within the host material, possibly creating localized magnetic regions. Additionally, the increased number of laser pulses might also induce changes in the oxidation states of dopant ions, altering their magnetic properties and increasing overall magnetization.

Figure 6a displays the hysteresis loop of the Ni:HAp thin film prepared with an initial number of 10,000 laser pulses. It was observed that at lower magnetic field values, the loop showed weak ferromagnetism, but at higher field values, the diamagnetic response of the Si substrate became dominant. The resulting diamagnetic curve, with a negative response to the external field, makes the material unsuitable for hyperthermia treatments. To address this limitation, a refined procedure was developed to deposit sufficient Ni:HAp on the Si substrate. One such solution was to increase the number of applied laser pulses. This consequently resulted in well-saturated hysteresis loops, as confirmed by magnetometer measurements. Usually, smaller ferromagnetic particles produce thinner hysteresis loops, whereas larger particles exhibit more pronounced ferromagnetic behavior with wider loops. The observed increase in saturation magnetization (M_s_) and remanent magnetization (M_r_) with an increased number of laser pulses is likely due to enhanced surface roughness.

The ferromagnetic behavior observed in the Ni:HAp thin films aligns with the presence of Ni within the HAp lattice. The nano-sized structure of Ni:HAp in this study demonstrated robust ferromagnetic properties, making it a promising candidate for use as thermo-seeds in cancer hyperthermia treatments. One should note here that the material’s ability to achieve controlled magnetic responses under varying conditions underscores its potential for biomedical applications, particularly in targeted cancer therapy.

The M-H curves, as depicted in Figure 6b,c, illustrate the ferromagnetic characteristics of the material based on the original data collected from all laser pulses. In contrast, Figure 6d highlights a distinct transformation in the magnetic moment, particularly within the M-H loop corresponding to the lowest and highest numbers of laser pulses. Key magnetic parameters, including M_s_, M_r_, coercivity (H_c_), and the squareness ratio of the hysteresis curve, were derived from the hysteresis loops. The inferred values corresponding to these parameters are summarized in Table 1.

The analysis revealed a monotonic increase in M_s_ and M_r_ values with the increasing number of laser pulses. Specifically, the M_s_ values vary from 2.57 × 10^−4^ emu to 4.87 × 10^−4^ emu for 35,000–60,000 laser pulses, while the M_r_ values range from 2.13 × 10^−4^ to 4.19 × 10^−4^ emu for the same laser pulse range. The trends for M_s_ and M_r_ as a function of the number of laser pulses are illustrated in Figure S2a,b. In contrast, the H_c_ values remain relatively constant, averaging 375 ± 25 Oe. Furthermore, the M_r_/M_s_ ratio calculated from the hysteresis data is presented in Figure S2c,d, providing additional insights into the magnetic behavior under varying laser pulse conditions.

### 2.7. Bioactivity

**Figure 7:**
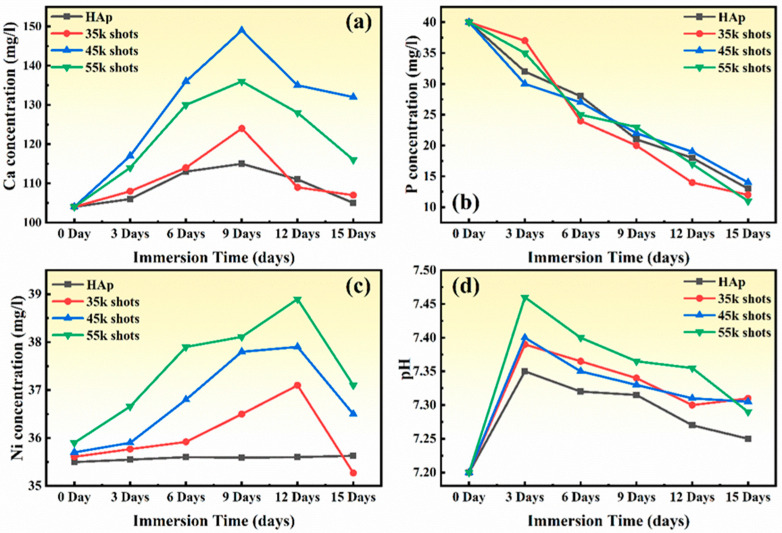
Bioactivity of HAp and Ni:HAp thin films after two weeks of immersion in simulated body fluid: effect of varying laser pulse numbers on (**a**) calcium (Ca), (**b**) phosphorus (P), and (**c**) nickel (Ni) concentrations, as well as (**d**) pH levels.

The bioactivity of the samples was evaluated using a simulated body fluid (SBF) solution, prepared according to the Kokubo protocol [42]. Figure 7a–d illustrate the variations in the concentrations of Ca, P, and Ni elements, as well as the pH, recorded intermittently over a two-week period during the immersion of the samples in SBF solution at 37 °C.

Initially, a rise in pH was observed (Figure 7d), which is likely attributed to the release of alkaline Ca^2+^ ions from the samples into the immersion medium. This is evidenced by the corresponding increase in Ca concentration (up to ~9 days) shown in Figure 7a. One should note that laser treatment modifies the surface morphology, introducing micro- and nanoscale features such as grooves or microcracks, which increase surface area and enhance dissolution. Additionally, samples treated with a higher number of laser pulses (Figure 7a) release more Ca, likely due to the increased intensity of surface restructuring and thermal effects, which enhance ion mobility and reactivity. After a 15-day immersion in SBF, ion concentration measurements revealed that the thin film produced with 55,000 laser pulses (Figure 7a) exhibited a Ca^2+^ ion concentration of 131 mg/L and a PO_4_^3−^ ion concentration of 13.76 mg/L. This may indicate reabsorption onto the surface, driven by supersaturation in the immersion medium.

The continuous decrease in P concentration is indicative of biomineralization processes, where P ions are consumed to form new CaP deposits on the surface. Samples fabricated with a higher number of laser pulses exhibit a steeper decline in P concentration. This could be attributed to the generation of a highly reactive surface with more nucleation sites due to thermally modified regions.

The increase in Ni concentration (Figure 7c) reflects Ni ion release from the substrate due to laser-induced surface modifications. Thermal effects and rapid cooling from laser treatment may create localized residual stresses or microstructural changes that facilitate Ni leaching. A higher number of laser pulses (Figure 7c) leads to more pronounced structural changes, increasing Ni release rates (37 mg/L of Ni^2+^ ions for the film fabricated with 45,000 pulses). The stabilization or decrease in Ni concentration after ~9–12 days may result from the formation of protective layers or depletion of readily leachable Ni at the surface.

Subsequently, the more reactive surface promotes faster ion release, resulting in a more pronounced pH increase (Figure 7d). After three days of immersion, a decrease in pH was also noted. This reduction is attributed to the formation of an apatite layer, which likely reduced Ca^2+^ ion leakage from the samples. In a physiological context, the formation of an apatite layer is influenced by the material’s resorbability. The pH of the solution corresponding to the film prepared with 35,000 laser pulses was recorded at 7.31, suggesting relatively lower resorbability for this sample. A higher number of laser pulses, which generates more reactive surfaces, exhibits larger initial pH increases and slower declines (Figure 7d) due to the extended activity of surface reactions. Thus, among the investigated samples, the Ni:HAp thin film deposited with 45,000 laser pulses exhibited the highest bioresorbability. This enhanced bioresorbability supports the suitability of this material for biomedical applications, particularly in contexts requiring controlled ion release.

**Figure 8:**
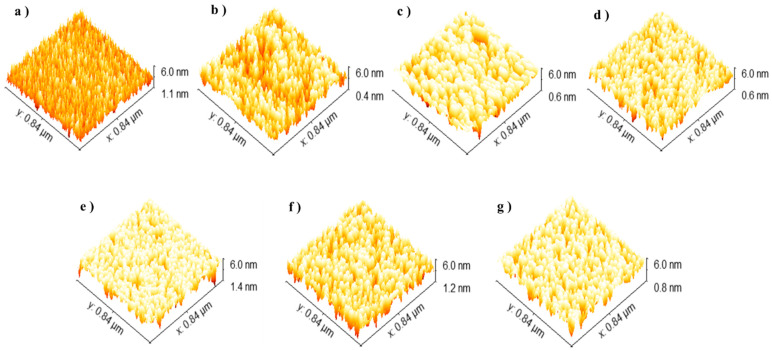
Surface morphologies analyzed by AFM: (**a**) image of HAp thin film surface after immersion and images of the Ni:HAp thin film surfaces treated with (**b**) 35,000 laser pulses prior to immersion, (**c**) 35,000 laser pulses after immersion, (**d**) 45,000 laser pulses prior to immersion, (**e**) 45,000 laser pulses after immersion, (**f**) 55,000 laser pulses before immersion, and (**g**) 55,000 laser pulses after immersion.

After all samples were removed from the SBF solution, their surface morphology was analyzed by AFM. Three-dimensional (3D) images were processed with Gwyddion software (version 2.60), providing detailed insights into the topographical features of the films, as shown in Figure 8.

These morphological analyses further confirm the relationship between laser pulse conditions, material properties, and bioresorbability, underscoring the potential of Ni:HAp thin films for advanced biomedical applications.

### 2.8. Protein–Ligand Interactions

**Figure 9:**
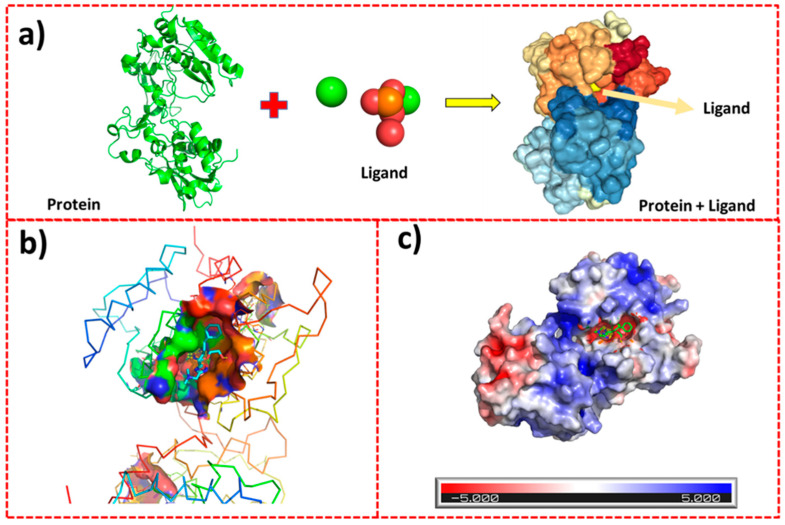
Ligand–protein interaction analysis: (**a**) interaction between the protein and ligand molecules, demonstrating that the ligand binds effectively to the protein; (**b**) key regions of the ligand–protein interaction highlighted, indicating a stable and persistent relationship; (**c**) electrostatic mapping, revealing crucial details about the stability and affinity of the binding site.

Figure 9a depicts the interaction between the protein and ligand molecules, illustrating the effective binding of the ligand to the protein. In particular, Figure 9b highlights the key regions involved in the ligand–protein interaction, emphasizing the stability and persistence of this relationship. The molecular docking analysis, conducted using the HDOCK platform, revealed a favorable binding affinity with a docking score of −67.73. The confidence score of 0.1617 suggests a reasonable level of reliability in the docking results. Additionally, the root mean square deviation of the ligand, calculated as 23.62 Å, quantifies the variation between the reference and predicted ligand positions, providing further insight into the binding conformation.

The structural alignment and binding predictions are supported by an LGscore of 4.32 and a MaxSub score of 0.281, indicating a high level of accuracy in the computational modeling. Electrostatic mapping provides crucial insights into the stability and affinity of the binding site, which are integral to understanding the ligand’s role in the biochemical process. Using the PyMOL gradient color scale, the visualization of electrostatic potential distributions ranging from −5 to +5 highlights key charge characteristics of the binding site.

Regions with high electron density or negative charge, typically near −5, appear in shades of red. These negatively charged areas are frequently associated with stabilizing electrostatic interactions with cationic species and are capable of attracting positively charged ions or molecules. Conversely, regions of electron deficiency or positive charge are near +5. These positively charged areas are often critical for substrate recognition, molecule binding, or protein–protein interactions, as they interact with negatively charged ligands or anionic functional groups. In this study, the negatively charged regions of the binding site interact with the guest molecule, facilitating robust electrostatic affinity. The presence of Ca^2+^ ions within the ligand molecules plays a pivotal role in enhancing these interactions. The Ca^2+^ ions contribute to achieving maximum electrostatic affinity between the ligand and receptor, as depicted in Figure 9c.

These findings emphasize the importance of electrostatic forces in ligand–receptor binding and provide a deeper understanding of the molecular interactions at play.

## 3. Discussion

To the best of our knowledge, only a limited number of articles have been published on Ni-substituted HAp fabricated by various techniques. The findings from some of these studies will be presented in the following paragraphs. Kim et al. recently synthesized nanotubes with sizes ranging from 20 to 160 nm and measured their coercive strength at RT. Their findings confirmed a single domain curve where super-paramagnetic behavior was observed in particles at a size of 20 nm, while particles with larger sizes deviated from this property [43]. Similarly, Krishnan and colleagues demonstrated that magnetic particles with a diameter of 16 nm exhibited a stronger response to an alternating magnetic field compared to larger particles, highlighting the size-dependent nature of magnetic behavior [44].

In our study, ferromagnetic particle sizes were found to range between 35 and 45 nm. These results align with recent findings by Baba and collaborators, who reported that magnetic particles with a larger diameter (i.e., 44 nm) exhibited improved heating efficiency in vitro compared to smaller particles in the 13–22 nm range [45]. According to their analysis, particles with diameters of 13 nm demonstrated super-paramagnetic properties, while those with diameters of 44 nm displayed ferromagnetic behavior. When applied to thermal therapy, both 13 nm and 44 nm nanoparticles were effective in suppressing MCF-7 cell lines. However, particles with a size of 44 nm induced a higher temperature increase in vitro than their 13 nm counterparts. This difference in temperature rise can be attributed to the coercive nature and enhanced coercive characteristics of the larger particles, which allow for more efficient heat generation under an alternating magnetic field.

There exists a diverse range of ferromagnetism-like crystalline materials with varying sizes. Among these, ferrite oxide is recognized as one of the most powerful natural magnetic minerals. It is commonly used in super-paramagnetic nanoparticles for various biological applications, including magnetic fluid hyperthermia (MFH) and MRI, as well as for delivering magnetic and genomic drugs [46]. For tissue-specific applications, particularly in targeting tumors, these nanoparticles are typically conjugated with tumor-specific ligands on their surfaces.

The surface modification of magnetic nanoparticles significantly alters their magnetic properties, primarily due to changes in anisotropy, which is crucial for hyperthermia applications. For instance, bioactive Fe-doped HAp synthesized by Tampieri et al. [47] has shown promising results. Similarly, Hou et al. reported in vivo hyperthermia outcomes for Fe-substituted HAp nanoparticles [48], while Murakami et al. developed magnetite/HAp composite materials via hydrothermal synthesis for bone cancer therapy [49].

The magnetic characteristics of Fe_2_O_3_ NPs are particularly challenging to control. In general, the super-paramagnetic behavior of such materials can only be tuned by altering the particle diameter. Unlike ferromagnetic materials, which generate heat under an AC magnetic field via a single mechanism at moderate to high frequencies, super-paramagnetic materials offer distinct thermal characteristics. Additionally, surface effects on nanoparticles, such as defect formation and surface stress [50,51], significantly impact their shape and functionality.

Magneto-mechanical effects have also been shown to enhance bone formation in bonded ferromagnetic fiber sequences [52]. Ferromagnetic bone cements have previously been developed for localized hyperthermia treatment within the skeletal system by combining magnetite and glass silicon powders with resin [53].

The treatment of bone fractures using magnetic therapy [54] has been previously explored and holds significant potential in the preparation and application of biomedical magnetic polymer composites [55]. In this study, the application of an alternating magnetic field to a ferromagnetic material induced movement of the magnetic domain walls. This movement, coupled with the reversal of magnetic moments as the field changes, generated heat losses in the material. The resulting heat production achieved the required temperature range for hyperthermia, highlighting the dual functionality of ferromagnetic materials in therapeutic applications. These properties are particularly valuable for developing advanced biomedical materials that integrate hyperthermia with structural support for bone regeneration.

The current study of Ni:HAp thin films deposited on Si substrates at varying numbers of laser pulses revealed critical properties that are advantageous for both biological and magnetic applications. The thin films exhibited a well-defined nanostructure with an average grain height of less than 2 nm. Magnetic force microscopy (MFM) confirmed the presence of magnetic particles on the surface, validating the magnetic character of the deposited Ni:HAp thin films.

In an alternating magnetic field, the sample produced with 45,000 laser pulses demonstrated the most favorable magnetic properties, characterized by the lowest squareness ratio and rapid heating performance. This sample achieved a temperature exceeding 45 °C within 80 s, meeting the critical threshold for cancer hyperthermia treatment. TGA analysis indicated a significant mass loss of ~46% at 800 °C, highlighting the material’s thermal decomposition behavior.

Bioactivity assessments revealed that the thin film prepared with 55,000 laser pulses released significant amounts of PO_4_^3−^ ions (13.76 mg/L) and Ca^2+^ ions (131 mg/L), which are essential for bone integration. The thin film fabricated with 45,000 laser pulses released 37 mg/L of Ni^2+^ ions, contributing to its magnetic properties. Additionally, the thin film prepared with 35,000 laser pulses exhibited the highest pH of 7.31 after 15 days of immersion in SBF, suggesting a stable and potentially biocompatible environment.

These findings highlight the functional potential of Ni:HAp thin films, combining magnetic properties suitable for hyperthermia treatment, bioactivity supportive of bone regeneration, and stability conducive to biomedical applications. This multifunctionality of Ni:HAp thin films positions them as promising candidates for the fabrication of advanced magnetic biomaterials.

## 4. Materials and Methods

### 4.1. Preparation of Ni:HAp Powders

The Ni:HAp nanoparticles were synthesized by the co-precipitation method, as described in Refs. [56,57]. The required chemicals were purchased from Merck, Germany. The synthesis process involved the preparation of Ni-substituted HAp (Ca_10−x_Ni_x_(PO_4_)_6_(OH)_2_) with a substitution level of Ni_x_ = 0.4. This was achieved by adding Ni (II) nitrate hexahydrate to a 0.05 M solution of calcium nitrate tetrahydrate (200 mL). The mixture was thoroughly stirred at a constant temperature of 80 °C to ensure uniform distribution and incorporation of the Ni ions into the HAp lattice. The synthesis of Ni powder was carried out by introducing a solution of diammonium hydrogen phosphate (0.03 M, 100 mL) and cetyltrimethylammonium bromide (CTAB, 0.03 M, 100 mL) drop-wise at a controlled rate of 1.5 mL/min to the reaction mixture. The pH of the solution was maintained within the range of 9–11 by adding ammonium hydroxide. The reaction was allowed to proceed overnight at room temperature (RT), facilitating the formation of precipitates. These precipitates were separated by centrifugation at 6000 rpm for 12 min, followed by two washes with distilled water to remove residual byproducts and impurities. The resulting gel-like material was subsequently dried at 50 °C for 48 h, resulting in the production of Ni:HAp powder. The as-obtained Ni:HAp powder was pressed into a 1-inch diameter target for use in the PLD experiments.

### 4.2. PLD Experiments

A Nd:YAG laser operating at a wavelength of 266 nm was used in the PLD experiments. The laser energy was set in the range of 100 mJ. The laser spot diameter was 1.45 mm, with a fluence of 6 J/cm^2^. The target-to-substrate separation distance was set to 50 mm, and the laser beam was adjusted to cover the entire target diameter. The chamber pressure was adjusted to 1.7 × 10^−6^ Torr, with the turbo molecular pump operating at a speed of 300 rpm. The laser beam was incident at 45° on the target’s surface. The number of laser pulses was varied between 30,000 and 60,000 (30,000 for HAp, and 35,000–60,000 for Ni:HAp thin films), with a Q switch delay of 235 μs. We would like to highlight that this specific range of laser pulses was selected based on preliminary adherence tests and prior experience. It was intentionally chosen to ensure optimal film thickness and uniformity for the Ni:HAp thin films while maintaining their structural integrity and critical functional properties. The target was rotated at a speed of 5°/s to ensure uniform deposition. Ni:HAp thin films were deposited on silicon substrates maintained at a constant temperature of 500 °C.

### 4.3. Characterization Techniques

The in vitro heat generation measurement was conducted to evaluate the thermal capacity of the as-obtained Ni:HAp, following previously established protocols with minor modifications [58,59,60]. The prepared samples were placed inside polypropylene bottles. The samples were cooled in a 1% agar solution to simulate biological conditions. A magnetic alternative field of 300 Oe at 100 kHz was applied to the polypropylene bottles containing the samples in agar gel. A fluor-optic thermometer was used to monitor temperature changes, providing data on the thermal output of the specimens under the applied magnetic field. In addition, thermogravimetric analysis (TGA) was performed using a DSC equipment (model Q200, TA Instruments, New Castle, DE, USA) to examine the thermal degradation behavior of the material. A sample weighing 6.5 mg was heated from 30 °C to 800 °C in a nitrogen atmosphere to prevent oxidation, with a gas flow rate of 60 mL/min. A constant heating rate of 10 °C/min was maintained throughout the analysis. The thermogravimetric curve was recorded to monitor weight loss and identify thermal transitions or decomposition events.

The structural analysis, surface topography, infrared, optical, and magnetic properties of the synthesized Ni:HAp thin films were investigated by XRD, AFM, FTIR, UV–visible spectroscopy, MFM, and M-H characteristics. Complementary heat generation assessment in an alternating magnetic field and TGA, along with protein–ligand interactions, were also performed.

The phase structure of the sample was analyzed using XRD with a Bruker D8 phaser instrument. The analysis was conducted using Cu Kα radiation, operating at 40 kV and a current of 200 mA.

The surface topography was evaluated using AFM in tapping mode.

FTIR analysis was conducted using a spectrometer (Thermo Fisher Scientific, Waltham, MA, USA) in the range of 4000 to 500 cm^−1^ to identify the functional groups present in the samples.

The morphological analysis was conducted using a FE-SEM microscope (model JSM7100F, Tokyo, Japan).

The magnetic properties of the Ni:HAp thin films were analyzed using a vibrating sample magnetometer (VSM) operated at a vibrational frequency of 30 Hz at RT. The hysteresis loop was measured within the range of +7kO_e_ to −7kO_e_ [61].

In vitro bioactivity tests were conducted by immersing the samples in SBF solution. After two weeks of immersion, the samples were removed from the SBF solution, carefully rinsed with 50 mL of distilled water followed by 20 mL of ethanol, and dried at RT. The surface morphology was subsequently analyzed using AFM, and 3D surface images were visualized using Gwyddion software.

In this study, the 6BTN protein (Bone Morphogenetic Protein 1 complexed with a reverse hydroxymate—compound **1**) was selected as the target due to its significant role in promoting the growth of cancer cells. The 6BTN protein structure was obtained from the Protein Data Bank (PDB) [62], and the ligand (HAp) was retrieved in CIF file format from the Crystallography Open Database [63] and converted to a PDB file using PyMOL software (version 3.04). Subsequently, the protein was imported into the molecular docking software AutoDock (version 1.5.7) for further analysis [64,65]. The protein was prepared for docking by removing water molecules, redundant chains, and heteroatoms to reduce interference during the simulations. To enhance hydrogen-bond interactions, polar hydrogens were added. Kollman charges were applied to the protein for accurate interaction modeling. The ligand molecule was uploaded into Auto-Dock Tools for preparation. Potential active sites on the target protein were identified using the HDOCK SERVER, a web-based molecular docking platform, to pinpoint regions critical for ligand interaction [66].

## 5. Conclusions

This study explores the effect of nickel doping in hydroxyapatite (Ni:HAp) thin films deposited on silicon (Si) substrates, with a focus on their optical, functional, morphological, and magnetic properties, as well as their crystal structure. The Ni:HAp thin films were synthesized using the pulsed laser deposition (PLD) technique, at varying numbers of laser pulses (i.e., in the range of 30,000–60,000). The findings indicate that an increase in the number of laser pulses corresponded to an increase in surface roughness. This effect was enhanced by the incorporation of Ni nanoparticles. A significant transition from diamagnetic to ferromagnetic behavior was observed, enhancing the material’s suitability for hyperthermia applications.

This research identified an optimized number of applied laser pulses for Ni:HAp thin films, enabling them to function as an efficient heat sink for self-regulated magnetic hyperthermia applications. With higher magnetization compared to other metal-based HAp nanoparticles, the Ni:HAp system achieved the optimal Curie temperature of 46 °C, making it effective for cancer hyperthermia treatment. These in vitro findings highlight the potential of Ni:HAp as a self-regulating material for future magnetic hyperthermia applications.

Additionally, docking studies and electrostatic mapping provided insights into the ligand–protein interactions, demonstrating a strong and stable relationship. The favorable docking score, complemented by key charge distributions, emphasizes the role of Ca^2+^ ions in enhancing electrostatic affinity, thereby facilitating effective binding. These results underline the importance of understanding ligand–protein interactions in advancing biochemical and biomedical applications.

While traditional studies have laid the groundwork by characterizing doped HAp materials, the lack of integrated in vitro hyperthermia investigations limits their translational potential in clinical settings. Future research should adopt a holistic approach, encompassing the synthesis, structural characterization, and thorough evaluation of hyperthermic properties under physiological conditions, alongside biocompatibility assessments. Such comprehensive studies are essential to harness the full therapeutic capabilities of doped-HAp-based materials in biomedical applications.

## Figures and Tables

**Table 1:** Saturation magnetization (M_s_), remanent magnetization (M_r_), coercivity (H_c_), and squareness ratio for Ni:HAp thin films prepared at varying numbers of laser pulses.

No. of Applied Laser Pulses	M_s_ (emu)	M_r_ (emu)	H_c_ (Oe)	M_r_/M_s_
35,000	2.57 × 10^−4^	2.14 × 10^−4^	367	0.83
40,000	2.59 × 10^−4^	1.96 × 10^−4^	300	0.76
45,000	3.65 × 10^−4^	2.59 × 10^−4^	385	0.71
50,000	3.076 × 10^−4^	2.35 × 10^−4^	371	0.77
55,000	3.10 × 10^−4^	2.63 × 10^−4^	365	0.85
60,000	4.88 × 10^−4^	4.19 × 10^−4^	394	0.86

## Data Availability

The original contributions presented in this study are included in the article and Supplementary Material. Further inquiries can be directed to the corresponding author.

## Supplementary Materials

The following supporting information can be downloaded at: https://www.mdpi.com/article/10.3390/ijms26031095/s1.

## References

[1] Zhang Y., Ding J., Qi B., Tao W., Wang J., Zhao C., Peng H., Shi J. (2019). Multifunctional Fibers to Shape Future Biomedical Devices. Adv. Funct. Mater..

[2] Wróblewska-Krepsztul J., Rydzkowski T., Michalska-Pożoga I., Thakur V.K. (2019). Biopolymers for Biomedical and Pharmaceutical Applications: Recent Advances and Overview of Alginate Electrospinning. Nanomaterials.

[3] Jhonsi M.A. (2018). Carbon Quantum Dots for Bioimaging. State of the Art in Nano-Bioimaging.

[4] Kalyane S. (2017). Basic of Nano Technology.

[5] Nanda S.S., Hembram K.P.S.S., Lee J.-K., Kim K., Selvan S.T., Yi D.K. (2019). Experimental and Theoretical Structural Characterization of Cu–Au Tripods for Photothermal Anticancer Therapy. ACS Appl. Nano Mater..

[6] Lotey G.S., Verma N.K. (2012). Structural, Magnetic, and Electrical Properties of Gd-Doped BiFeO_3_ Nanoparticles with Reduced Particle Size. J. Nanoparticle Res..

[7] Fiume E., Magnaterra G., Rahdar A., Verné E., Baino F. (2021). Hydroxyapatite for Biomedical Applications: A Short Overview. Ceramics.

[8] Singh G., Singh R.P., Jolly S.S. (2020). Customized Hydroxyapatites for Bone-Tissue Engineering and Drug Delivery Applications: A Review. J. Sol-Gel Sci. Technol..

[9] Renaudin G., Gomes S., Nedelec J.-M. (2017). First-Row Transition Metal Doping in Calcium Phosphate Bioceramics: A Detailed Crystallographic Study. Materials.

[10] Inam H., Sprio S., Tavoni M., Abbas Z., Pupilli F., Tampieri A. (2024). Magnetic Hydroxyapatite Nanoparticles in Regenerative Medicine and Nanomedicine. Int. J. Mol. Sci..

[11] Sedighi O., Alaghmandfard A., Montazerian M., Baino F. (2022). A Critical Review of Bioceramics for Magnetic Hyperthermia. J. Am. Ceram. Soc..

[12] Brannon-Peppas L., Blanchette J.O. (2004). Nanoparticle and Targeted Systems for Cancer Therapy. Adv. Drug Deliv. Rev..

[13] Neophytou C.M., Panagi M., Stylianopoulos T., Papageorgis P. (2021). The Role of Tumor Microenvironment in Cancer Metastasis: Molecular Mechanisms and Therapeutic Opportunities. Cancers.

[14] Mantyh P. (2013). Bone Cancer Pain: Causes, Consequences, and Therapeutic Opportunities. Pain.

[15] Slaoui A., Albisinni S., Aoun F., Assenmacher G., Al Hajj Obeid W., Diamand R., Regragui S., Touzani A., Bakar A., Mesfioui A. (2019). A Systematic Review of Contemporary Management of Oligometastatic Prostate Cancer: Fighting a Challenge or Tilting at Windmills?. World J. Urol..

[16] Cifrić S., Spahić Bećirović L., Osmanović D., Imamović E., Deumić A., Badnjevic A., Gurbeta Pokvić L. (2021). Fighting Cancer Using Nanoparticles—Diagnosis, Treatment and Monitoring. Proceedings of the International Conference on Medical and Biological Engineering.

[17] Logothetis C., Morris M.J., Den R., Coleman R.E. (2018). Current Perspectives on Bone Metastases in Castrate-Resistant Prostate Cancer. Cancer Metastasis Rev..

[18] Lin S.-C., Yu-Lee L.-Y., Lin S.-H. (2018). Osteoblastic Factors in Prostate Cancer Bone Metastasis. Curr. Osteoporos. Rep..

[19] Wust P., Hildebrandt B., Sreenivasa G., Rau B., Gellermann J., Riess H., Felix R., Schlag P. (2002). Hyperthermia in Combined Treatment of Cancer. Lancet Oncol..

[20] Liu X., Zhang Y., Wang Y., Zhu W., Li G., Ma X., Zhang Y., Chen S., Tiwari S., Shi K. (2020). Comprehensive Understanding of Magnetic Hyperthermia for Improving Antitumor Therapeutic Efficacy. Theranostics.

[21] Bromma K., Chithrani D.B. (2020). Advances in Gold Nanoparticle-Based Combined Cancer Therapy. Nanomaterials.

[22] Mondal S., Manivasagan P., Bharathiraja S., Santha Moorthy M., Nguyen V.T., Kim H.H., Nam S.Y., Lee K.D., Oh J. (2017). Hydroxyapatite Coated Iron Oxide Nanoparticles: A Promising Nanomaterial for Magnetic Hyperthermia Cancer Treatment. Nanomaterials.

[23] Ramos-Guivar J.A., Morales M.A., Litterst F.J. (2020). γ-Fe_2_O_3_ Nanoparticles Embedded in Nanohydroxyapatite Matrix for Magnetic Hyperthermia and in Vitro Osteoblast Cell Studies. Ceram. Int..

[24] Jiang Y., Ou J., Zhang Z., Qin Q.-H. (2011). Preparation of Magnetic and Bioactive Calcium Zinc Iron Silicon Oxide Composite for Hyperthermia Treatment of Bone Cancer and Repair of Bone Defects. J. Mater. Sci. Mater. Med..

[25] Laurent S., Dutz S., Häfeli U.O., Mahmoudi M. (2011). Magnetic Fluid Hyperthermia: Focus on Superparamagnetic Iron Oxide Nanoparticles. Adv. Colloid Interface Sci..

[26] Ciobanu C.S., Nica I.C., Dinischiotu A., Iconaru S.L., Chapon P., Bita B., Trusca R., Groza A., Predoi D. (2022). Novel Dextran Coated Cerium Doped Hydroxyapatite Thin Films. Polymers.

[27] Duta L., Stan G.E., Popescu-Pelin G., Zgura I., Anastasescu M., Oktar F.N. (2022). Influence of Post-Deposition Thermal Treatments on the Morpho-Structural, and Bonding Strength Characteristics of Lithium-Doped Biological-Derived Hydroxyapatite Coatings. Coatings.

[28] Duta L. (2021). In Vivo Assessment of Synthetic and Biological-Derived Calcium Phosphate-Based Coatings Fabricated by Pulsed Laser Deposition: A Review. Coatings.

[29] Kylychbekov S., Allamyradov Y., Khuzhakulov Z., Majidov I., Banga S., ben Yosef J., Duta L., Er A.O. (2023). Bioactivity and Mechanical Properties of Hydroxyapatite on Ti6Al4V and Si(100) Surfaces by Pulsed Laser Deposition. Coatings.

[30] El-Kader M.F.H.A., Ahmed M.K., Elabbasy M.T., Afifi M., Menazea A.A. (2021). Morphological, Ultrasonic Mechanical and Biological Properties of Hydroxyapatite Layers Deposited by Pulsed Laser Deposition on Alumina Substrates. Surf. Coat. Technol..

[31] Chunarkar-Patil P., Kaleem M., Mishra R., Ray S., Ahmad A., Verma D., Bhayye S., Dubey R., Singh H.N., Kumar S. (2024). Anticancer Drug Discovery Based on Natural Products: From Computational Approaches to Clinical Studies. Biomedicines.

[32] Li H., Sun X., Cui W., Xu M., Dong J., Ekundayo B.E., Ni D., Rao Z., Guo L., Stahlberg H. (2024). Computational Drug Development for Membrane Protein Targets. Nat. Biotechnol..

[33] Niu X., Yuan M., Zhao R., Wang L., Liu Y., Zhao H., Li H., Yang X., Wang K. (2024). Fabrication Strategies for Chiral Self-Assembly Surface. Microchim. Acta.

[34] Ahmed L.O., Omer R.A. (2024). Hydroxyapatite Biomaterials: A Comprehensive Review of Their Properties, Structures, Clinical Applications, and Producing Techniques. Rev. Inorg. Chem..

[35] Kurinjinathan P., Thanigai Arul K., Ramana Ramya J., Manikandan E., Hegazy H.H., Umar A., Algarni H., Ahmad N. (2020). Effect of Nickel Doping on the Properties of Hydroxyapatite Nanoparticles. J. Nanosci. Nanotechnol..

[36] Tithito T., Sillapaprayoon S., Chantho V., Pimtong W., Thongbunchoo J., Charoenphandhu N., Krishnamra N., Yong N., Lert-Itthiporn A., Maneeprakorn W. (2024). Evaluation of Magnetic Hyperthermia, Drug Delivery and Biocompatibility (Bone Cell Adhesion and Zebrafish Assays) of Trace Element Co-Doped Hydroxyapatite Combined with Mn–Zn Ferrite for Bone Tissue Applications. RSC Adv..

[37] Kamitakahara M., Ohtoshi N., Kawashita M. (2016). Spherical Porous Hydroxyapatite Granules Containing Composites of Magnetic and Hydroxyapatite Nanoparticles for the Hyperthermia Treatment of Bone Tumor. J. Mater. Sci. Mater. Med..

[38] Li B., Yuan X., Li B., Wang X. (2020). Impact of Pore Structure on Hydroxyapatite Supported Nickel Catalysts (Ni/HAP) for Dry Reforming of Methane. Fuel Process. Technol..

[39] Gibson I.R., Best S.M., Bonfield W. (1999). Chemical Characterization of Silicon-Substituted Hydroxyapatite. J. Biomed. Mater. Res. Off. J. Soc. Biomater. Jpn. Soc. Biomater. Aust. Soc. Biomater..

[40] Alshemary A.Z., Akram M., Goh Y.-F., Tariq U., Butt F.K., Abdolahi A., Hussain R. (2015). Synthesis, Characterization, In Vitro Bioactivity and Antimicrobial Activity of Magnesium and Nickel Doped Silicate Hydroxyapatite. Ceram. Int..

[41] Ider M., Abderrafi K., Eddahbi A., Ouaskit S., Kassiba A. (2017). Silver Metallic Nanoparticles with Surface Plasmon Resonance: Synthesis and Characterizations. J. Clust. Sci..

[42] Kokubo T., Kushitani H., Sakka S., Kitsugi T., Yamamuro T. (1990). Solutions Able to Reproducein Vivo Surface-Structure Changes in Bioactive Glass-Ceramic A-W3. J. Biomed. Mater. Res..

[43] Kim D., Lee N., Park M., Kim B.H., An K., Hyeon T. (2009). Synthesis of Uniform Ferrimagnetic Magnetite Nanocubes. J. Am. Chem. Soc..

[44] Khandhar A.P., Ferguson R.M., Krishnan K.M. (2011). Monodispersed Magnetite Nanoparticles Optimized for Magnetic Fluid Hyperthermia: Implications in Biological Systems. J. Appl. Phys..

[45] Baba D., Seiko Y., Nakanishi T., Zhang H., Arakaki A., Matsunaga T., Osaka T. (2012). Effect of Magnetite Nanoparticles on Living Rate of MCF-7 Human Breast Cancer Cells. Colloids Surf. B Biointerfaces.

[46] Mody V.V., Singh A., Wesley B. (2013). Basics of Magnetic Nanoparticles for Their Application in the Field of Magnetic Fluid Hyperthermia. Eur. J. Nanomed..

[47] Tampieri A., D’Alessandro T., Sandri M., Sprio S., Landi E., Bertinetti L., Panseri S., Pepponi G., Goettlicher J., Bañobre-López M. (2012). Intrinsic Magnetism and Hyperthermia in Bioactive Fe-Doped Hydroxyapatite. Acta Biomater..

[48] Hou C.-H., Hou S.-M., Hsueh Y.-S., Lin J., Wu H.-C., Lin F.-H. (2009). The in Vivo Performance of Biomagnetic Hydroxyapatite Nanoparticles in Cancer Hyperthermia Therapy. Biomaterials.

[49] Murakami S., Hosono T., Jeyadevan B., Kamitakahara M., Ioku K. (2008). Hydrothermal Synthesis of Magnetite/Hydroxyapatite Composite Material for Hyperthermia Therapy for Bone Cancer. J. Ceram. Soc. Jpn..

[50] Farzin A., Fathi M., Emadi R. (2017). Multifunctional Magnetic Nanostructured Hardystonite Scaffold for Hyperthermia, Drug Delivery and Tissue Engineering Applications. Mater. Sci. Eng. C.

[51] Veverka P., Pollert E., Závěta K., Vasseur S., Duguet E. (2008). Sr-Hexaferrite/Maghemite Composite Nanoparticles—Possible New Mediators for Magnetic Hyperthermia. Nanotechnology.

[52] Markaki A.E., Clyne T.W. (2004). Magneto-Mechanical Stimulation of Bone Growth in a Bonded Array of Ferromagnetic Fibres. Biomaterials.

[53] Takegami K., Sano T., Wakabayashi H., Sonoda J., Yamazaki T., Morita S., Shibuya T., Uchida A. (1998). New Ferromagnetic Bone Cement for Local Hyperthermia. J. Biomed. Mater. Res..

[54] Baibekov I.M., Khanapiyaev U.K. (2001). Healing of Bone Fractures of Rat Shin and Some Immunological Indices during Magnetic Laser Therapy and Osteosynthesis by the Ilizarov Method. Bull. Exp. Biol. Med..

[55] Keshri S., Kumar V., Wiśniewski P., Kamzin A.S. (2014). Synthesis and Characterization of LSMO Manganite-Based Biocomposite. Phase Transit..

[56] Asghar M.S., Li J., Ahmed I., Ghazanfar U., Irshad M.S., Idrees M., Haq Z., Rizwan M., Sheikh F., Yasmeen F. (2021). Antioxidant, and Enhanced Flexible Nano Porous Scaffolds for Bone Tissue Engineering Applications. Nano Sel..

[57] Asghar M.S., Ghazanfar U., Idrees M., Irshad M.S., Haq Z., Javed M.Q., Hassan S.Z., Rizwan M. (2023). In Vitro Controlled Drug Delivery of Cationic Substituted Hydroxyapatite Nanoparticles; Enhanced Anti-Chelating and Antibacterial Response. Kuwait J. Sci..

[58] Li Z., Kawashita M., Araki N., Mitsumori M., Hiraoka M., Doi M. (2011). Preparation of Magnetic Iron Oxide Nanoparticles for Hyperthermia of Cancer in a FeCl_2_-NaNO_3_-NaOH Aqueous System. J. Biomater. Appl..

[59] Li Z., Kawashita M., Araki N., Mitsumori M., Hiraoka M., Doi M. (2010). Magnetite Nanoparticles with High Heating Efficiencies for Application in the Hyperthermia of Cancer. Mater. Sci. Eng. C.

[60] Kawashita M., Domi S., Saito Y., Aoki M., Ebisawa Y., Kokubo T., Saito T., Takano M., Araki N., Hiraoka M. (2008). In Vitro Heat Generation by Ferrimagnetic Maghemite Microspheres for Hyperthermic Treatment of Cancer under an Alternating Magnetic Field. J. Mater. Sci. Mater. Med..

[61] Krebs H.-U., Weisheit M., Faupel J., Süske E., Scharf T., Fuhse C., Störmer M., Sturm K., Seibt M., Kijewski H., Kramer B. (2003). Pulsed Laser Deposition (PLD)—A Versatile Thin Film Technique BT—Advances in Solid State Physics. Advances in Solid State Physics.

[62] Gampe R., Shewchuk L. (2024). BMP1 Complexed with a Reverse Hydroxymate—Compound 1. https://www.wwpdb.org/pdb?id=pdb_00006btn.

[63] Ardanova L.I., Get’man E.I., Loboda S.N., Prisedsky V.V., Tkachenko T.V., Marchenko V.I., Antonovich V.P., Chivireva N.A., Chebishev K.A., Lyashenko A.S. (2010). Isomorphous Substitutions of Rare Earth Elements for Calcium in Synthetic Hydroxyapatites. Inorg. Chem..

[64] Trott O., Olson A.J. (2010). AutoDock Vina: Improving the Speed and Accuracy of Docking with a New Scoring Function, Efficient Optimization, and Multithreading. J. Comput. Chem..

[65] Seeliger D., de Groot B.L. (2010). Ligand Docking and Binding Site Analysis with PyMOL and Autodock/Vina. J. Comput. Aided. Mol. Des..

[66] Yan Y., Tao H., He J., Huang S.-Y. (2020). The HDOCK Server for Integrated Protein–Protein Docking. Nat. Protoc..

