# Supplementary material for: Potential Molecular Interactions and In Vitro Hyperthermia, Thermal, and Magnetic Studies of Bioactive Nickel-Doped Hydroxyapatite Thin Films
Source: Int J Mol Sci. 2025 Jan 27;26(3):1095. doi: 10.3390/ijms26031095 (PMC11817106; doi:10.3390/ijms26031095)
Supplement: Supplementary file 1 [file ijms-26-01095-s001.zip › ijms-3403398-supplementary.pdf]

Table S1. Elemental compositions from EDS mapping of Ni:HAp thin films.

Element	Concentration [wt.%]
Oxygen	70.09
Calcium	19.26
Phosphorous	9.63
Nickel	1.02

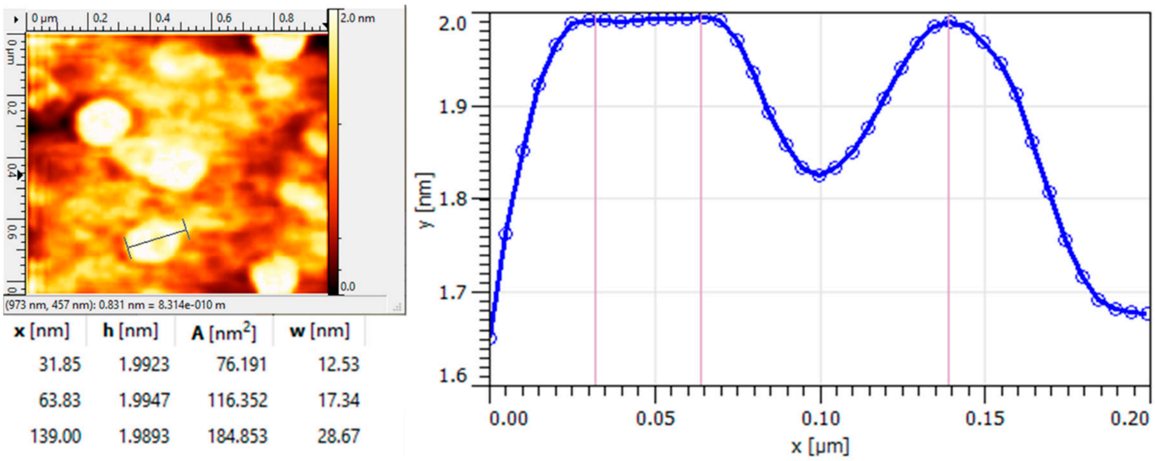

Figure S1. Line analysis of the doughnut-shaped particulates observed on the surface of Ni:HAp thin films.

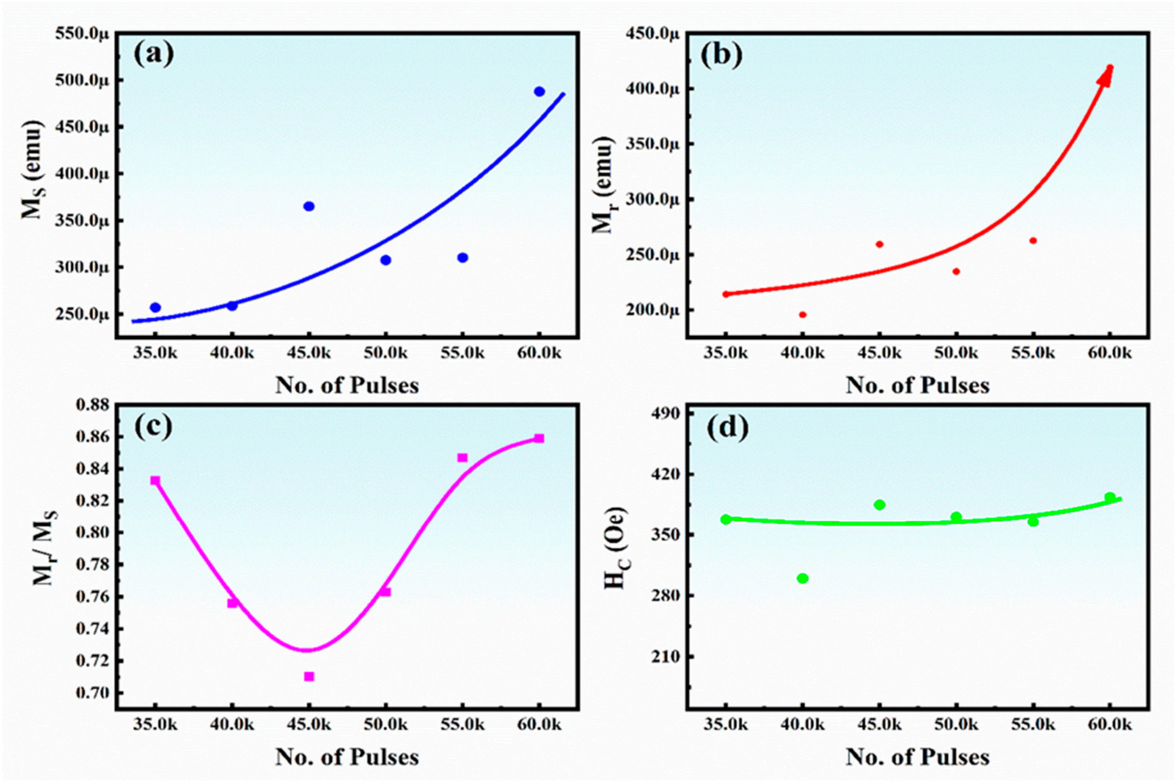

Figure S2. The curves of (a) Saturation Magnetization (M_s), (b) Remanence Magnetization (M_r), (c) Squareness ratio (M_r/M_s), and (d) Coercivity (H_c) as functions of the number of applied laser pulses for Ni:HAp thin films.
